# Predictors of cardiac rehabilitation referral, enrolment and completion after acute myocardial infarction: an exploratory study

**DOI:** 10.1007/s12471-020-01492-0

**Published:** 2020-10-08

**Authors:** R. W. M. Brouwers, V. J. G. Houben, J. J. Kraal, R. F. Spee, H. M. C. Kemps

**Affiliations:** Flow, Center for Prevention, Telemedicine and Rehabilitation in Chronic Disease, Máxima Medical Center, Veldhoven/Eindhoven, The Netherlands

**Keywords:** Cardiac rehabilitation, Acute myocardial infarction, Referral, Enrolment, Participation, Completion

## Abstract

**Background:**

Despite proven clinical benefits, only a minority of patients complete outpatient cardiac rehabilitation (CR) after acute myocardial infarction (AMI). The main purpose of this study was to evaluate to what extent and at which time patients drop out of CR, and to assess which patient-related characteristics can predict dropout.

**Methods:**

In a retrospective cohort study, we selected patients who had been hospitalised with an AMI in our centre in 2015 or 2016. Patients were selected pseudonymously based on reimbursement codes in the electronic health record. We extracted baseline characteristics and data on CR referral, enrolment and completion for each patient. Multivariable logistic regression was used to assess which characteristics predicted referral and dropout.

**Results:**

The 666 patients included were predominantly male (66%), with a mean age of 69.0 years. Of the 640 eligible patients, 201 (31%) were not referred for CR. Enrolment after referral was 94%. Nonreferral was independently associated with older age, female sex, traveling distance, non-ST-elevation myocardial infarction (NSTEMI; as compared with STEMI), no coronary revascularisation and prior manifestations of coronary artery disease. Of the 414 enrolled patients, 24% did not complete their CR programmes (i.e. dropped out). Older age and worse exercise capacity at baseline were independently associated with dropout. The ability of the multiple regression models to predict nonreferral and noncompletion was good to fair, with an area under the receiver operating characteristic curves of 0.86 and 0.71, respectively.

**Conclusion:**

The main reason for not participating in or not completing CR after AMI was nonreferral. To optimise CR utilisation, improvement of referral rates should be prioritised.

## What’s new?

- The main reason for not participating in or not completing cardiac rehabilitation (CR) after acute myocardial infarction was nonreferral.
- Although dropout after referral was lower, more than half of discharged patients ultimately did not complete a CR programme.
- To optimise CR utilisation, improvement of referral rates should have priority, focusing on elderly patients and women, and on patients with non-ST-elevation myocardial infarction, without coronary revascularisation or with prior manifestations of coronary artery disease.
- Alternative models of CR, such as cardiac telerehabilitation, should be considered to improve CR enrolment and completion.

## Introduction

Cardiac rehabilitation (CR) reduces cardiovascular morbidity and mortality and increases quality of life in patients after acute myocardial infarction (AMI) [1–3]. Therefore, CR is strongly recommended in American and European guidelines [4–7]. Still, less than half of eligible patients with AMI or other manifestations of coronary artery disease (CAD) participate in outpatient CR [8]. Low participation rates are caused by suboptimal referral by medical professionals and unsatisfactory enrolment of patients after referral [9]. After enrolment, up to one-third of participants do not complete their CR programmes [10]. Even though low CR participation and completion rates are well studied and improved uptake has been proven beneficial in cost-benefit analyses [11, 12], detailed recommendations on strategies to improve these rates are lacking in current guidelines.

Not participating or not completing CR is associated with a wide range of factors at the patient level, healthcare professional level and (healthcare) system level [13, 14]. Examples of these factors include demographic or disease-specific factors (patient level), physician CR endorsement (professional level) and financial or geographic factors (system level). While not participating and dropout of CR are associated with a doubled risk for cardiovascular events or death [15], nonparticipation and dropout are more often seen in older and high risk patients (i.e. patients with cardiovascular risk factors or lower socioeconomic status) [16–18]. As this may even further increase their cardiovascular risks, it is of eminent importance to understand why and at which times during a CR programme these patients drop out.

Despite a growing body of evidence on interventions to increase CR utilisation, the percentage of participating patients has not increased in Europe in the past 7 years [8]. To identify the weakest link in the CR care pathway—and thus the primary target for improvement—it is important to chart the entire pathway, and not to solely focus on CR referral or completion. Therefore, the objective of this study was to evaluate at which time (from hospital discharge to CR completion) patients drop out of CR, and to assess which patient-related characteristics can predict dropout.

## Methods

### Study design

In this retrospective cohort study, we selected patients who had been hospitalised with an AMI in our Department of Cardiology between 1 January 2015 and 31 December 2016. Patients were selected pseudonymously based on reimbursement codes in the electronic health record (EHR) and data were extracted using the EHR Data Platform version 1.2.10 from CTcue (Amsterdam, the Netherlands) [19]. The study design was approved by the Medical Ethics Review Committee of Máxima Medical Center Veldhoven, the Netherlands.

### Study population and follow-up

We selected patients who had been hospitalised in our centre with a non-ST-elevation myocardial infarction (NSTEMI) or ST-elevation myocardial infarction (STEMI). Patients who already participated in outpatient CR at the time of hospitalisation, were excluded from all analyses, as this would likely influence participation in a new CR programme. Follow-up after the index event (i.e. hospitalisation for AMI) was maximised at 12 months, to minimise the possibility of incorrectly attributing components of a second CR programme to the index event.

### Cardiac rehabilitation programme

During the study period, patients with AMI were semiautomatically referred for CR at the time of hospital discharge by the treating physician via the EHR. When the EHR registered that a patient was hospitalised with an AMI, the physician received an automatic notification that CR was advised for that patient. At discharge, the physician then created a partly prefilled CR referral form in the EHR, containing relevant clinical characteristics, in order to refer the patient. This semiautomatic referral strategy was combined with advice (liaison) by nurses on the ward.

Following the current Dutch CR guideline [20], patients referred for CR after AMI were offered an individualised outpatient programme of 12 weeks, consisting of one or more group-based therapies (i.e. exercise training, education, relaxation therapy, psychoeducative prevention therapy, smoking cessation therapy) and/or individual treatment by a psychologist, dietician or social worker. The content of a patient’s programme was based on an individual needs assessment [21]. After 3 months, the programme was evaluated at the outpatient clinic.

### Data extraction

For each patient, demographic, geographic and clinical characteristics were collected at baseline (i.e. hospitalisation or, if applicable, start of the CR programme). For patients attending the programme, data from the individual needs assessment [21] were also collected. This included data on exercise testing, marital and employment status, and questionnaires on health-related quality of life (*Kwaliteit van Leven bij Hartpatiënten*, a validated Dutch translation of the MacNew Heart Disease Health-Related Quality of Life Questionnaire), anxiety (Generalized Anxiety Disorder 7‑item Scale) and depression (Patient Health Questionnaire-9) [21].

### Outcome measures

Outcome measures were CR referral, enrolment (attending the intake procedure), attendance at one or more therapies, and programme completion (attending the evaluation procedure after 12 weeks).

### Statistical analysis

Continuous variables are reported as mean ± standard deviation when normally distributed or as median and interquartile range when not normally distributed. Categorical variables are presented as number and percentage. If the presence or absence of cardiovascular risk factors or prior manifestations of CAD was not documented (i.e. data were missing), we assumed they were absent. We analysed differences in baseline characteristics between patients for the outcome measures using the chi-squared test or (if the expected cell count for a variable was less than five) the Fisher’s exact test for categorical variables, and the independent samples *t*-test or (if the variable was not normally distributed) the Mann-Whitney U test for continuous variables. Statistical tests were two-tailed, and *p*-values were considered statistically significant when *p* < 0.05.

Variables for which a statistically significant between-group difference (*p* < 0.05) was found in univariate analysis, were used in multivariable logistic regression analyses to assess which variables were associated with the outcome measures (complete-case analysis). Because of the explorative design of the study, we used backwards stepwise multivariable logistic regression to calculate the odds ratio and 95% confidence interval for each variable. For each multivariable model, we plotted a receiver operating characteristic (ROC) curve and calculated the area under the curve to assess its discriminative ability. Statistical analyses were performed using SPSS Statistics version 22 (IBM Corp. Armonk, NY, USA).

## Results

In 2015 and 2016, 666 patients were hospitalised with a NSTEMI (65%) or STEMI (35%). Patients were predominantly male (66%), with a mean age of 69.0 ± 12.8 years. Twenty patients died during hospitalisation and 6 already participated in outpatient CR at the time of hospitalisation. Data of the remaining 640 patients were analysed.

### Dropout during CR

**Fig. 1:**
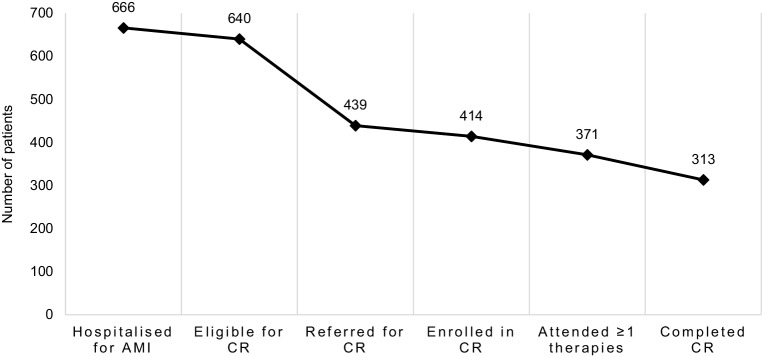
Nonparticipation and dropout during cardiac rehabilitation. *AMI* acute myocardial infarction, *CR* cardiac rehabilitation

In total, 201 out of the 640 included patients (31%) were not referred for CR. Of the 439 referred patients, 25 (6%) did not enrol. Of the 414 enrolled patients, 43 (10%) did not attend any therapies, and of the 371 patients attending one or more therapies, 58 (16%) did not attend the evaluation procedure. A total of 313 patients (49% of eligible patients and 71% of those referred) completed a CR programme (Fig. 1).

### Predictors of referral

**Fig. 2:**
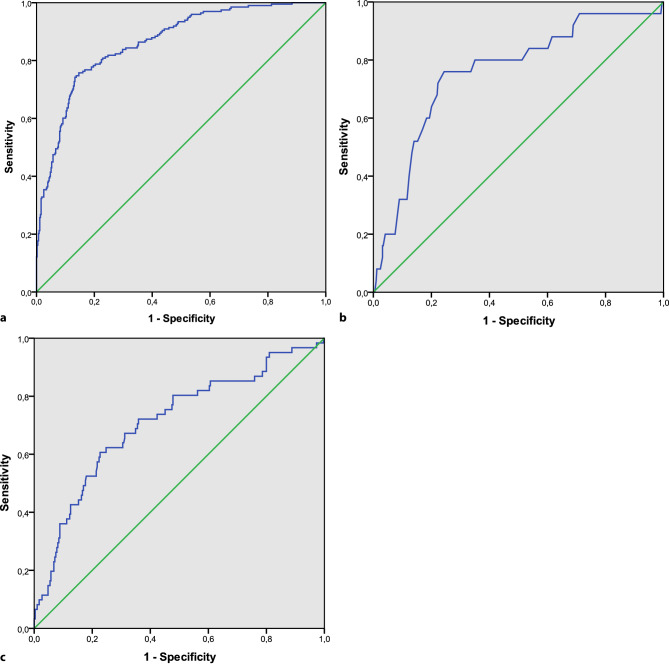
Receiver operating characteristic curves for multivariable models, predicting **a** referral, **b** enrolment and **c** completion

Patients who were not referred for CR were older, more often female, had a longer traveling distance to the CR centre, more often presented with NSTEMI (as compared with STEMI), were less often revascularised and more often had prior manifestations of CAD (Tab. 1). All these variables were independently associated with nonreferral (Tab. 2). The chance of nonreferral was 1.7 times higher in women, 2.2 times higher for patients with NSTEMI and 11 times higher for patients who were not revascularised. The area under the ROC curve for the multiple regression model including these variables was 0.86, indicating good discriminatory ability (Fig. 2a).

**Table 1:** Baseline characteristics according to CR referral and completion

		Referred			Completed		
Variable		No (*n* = 201)	Yes (*n* = 439)	*P*-value	No (*n* = 101)	Yes (*n* = 313)	*P*-value
Men		108 (54%)	316 (72%)	<0.001	66 (65%)	234 (75%)	0.066
Age at hospitalisation, years		74.1 ± 12.7	66.1 ± 12.1	<0.001	71.2 ± 10.8	63.8 ± 11.7	<0.001
Age category, years	<40	2 (1%)	7 (2%)			6 (2%)	
	40–49	9 (4%)	49 (11%)		7 (7%)	42 (13%)	
	50–59	17 (8%)	84 (19%)		10 (10%)	73 (23%)	
	60–69	42 (21%)	122 (28%)		24 (24%)	92 (29%)	
	70–79	60 (30%)	128 (29%)		39 (39%)	81 (26%)	
	≥80	71 (35%)	49 (11%)		21 (21%)	19 (6%)	
Presentation	NSTEMI	169 (84%)	245 (56%)	<0.001	58 (57%)	170 (54%)	0.585
	STEMI	32 (16%)	194 (44%)		43 (43%)	143 (46%)	
Coronary revascularisation	Yes	32 (16%)	309 (70%)	<0.001	69 (68%)	227 (73%)	0.415
	PCI	29 (14%)	258 (59%)		56 (55%)	189 (60%)	
	CABG	3 (1%)	51 (12%)		13 (13%)	38 (12%)	
	No	169 (84%)	130 (30%)		32 (32%)	86 (27%)	
Distance to hospital, km		15 (22.5)	13.8 (14.3)	0.036	13.5 (11.1)	14 (18.4)	0.133
Admission length, days		4 (5)	4 (4)	0.580	4 (4)	4 (3)	0.042
Waiting time, days					12 (12)	10 (11)	0.057
BMI, kg/m^2^		26.2 (6.9)	26.5 (5.1)	0.213	26 (4.7)	26.8 (5)	0.089
Hypertension					58 (57%)	144 (46%)	0.046
Hypercholesterolaemia					68 (67%)	237 (76%)	0.096
Smoking at hospitalisation					26 (26%)	103 (33%)	0.176
Diabetes mellitus					25 (25%)	38 (12%)	0.002
Positive family history for CAD					47 (47%)	177 (57%)	0.079
Documented history of CAD		101 (50%)	124 (28%)	<0.001	40 (40%)	72 (23%)	0.001
Previous CR participation		16 (8%)	16 (4%)	0.020	4 (4%)	12 (4%)	1.000
Maximal workload, % of expected workload					78.6 ± 27	90.4 ± 21.6	0.002
Living together or married					30 (71%)	173 (80%)	0.232
Employed					10 (11%)	118 (41%)	<0.001
KvL-H: total score					4.9 ± 1.1	5.2 ± 1	0.049
KvL-H: emotional score					4.9 ± 1.2	5.1 ± 1.1	0.129
KvL-H: physical score					4.6 ± 1.3	4.9 ± 1.2	0.030
KvL-H: social score					5.2 ± 1.2	5.5 ± 1.1	0.023
PHQ‑2 score					1 (0)	0 (0)	0.203
PHQ‑9 score					4 (1)	3 (1)	0.299
GAD‑7 score					2 (0)	2 (0)	0.620

Values are reported as *n *(%), mean ± standard deviation, or median (interquartile range)

*CR* cardiac rehabilitation, *NSTEMI* non-ST-elevation myocardial infarction, *STEMI* ST-elevation myocardial infarction, *PCI* percutaneous coronary intervention, *CABG* coronary artery bypass grafting, *BMI* body mass index, *CAD* coronary artery disease, *KvL‑H* validated Dutch translation of MacNew Heart Disease Health-Related Quality of Life Questionnaire (*Kwaliteit van Leven bij Hartpatiënten*), *PHQ* Patient Health Questionnaire, *GAD* Generalized Anxiety Disorder Scale

**Table 2:** Multivariable predictors of nonreferral and dropout (noncompletion) for CR

	Nonreferral			Dropout		
Predictor	OR	95% CI	*P*-value	OR	95% CI	*P*-value
Female sex	1.70	1.09–2.66	0.020			
Age at hospitalisation, per year	1.04	1.02–1.06	<0.001	1.05	1.02–1.08	0.002
NSTEMI	2.24	1.35–3.72	0.002			
No coronary revascularisation	11.06	6.93–17.64	<0.001			
Distance to CR centre, per km	1.03	1.01–1.04	0.003			
Prior manifestation of CAD	2.20	1.39–3.49	0.001			
Maximal workload, per 10% of expected workload				0.81	0.70–0.93	0.004

*CR* cardiac rehabilitation, *OR* odds ratio, *CI* confidence interval, *NSTEMI* non-ST-elevation myocardial infarction, *CAD* coronary artery disease

### Predictors of enrolment

Of the referred patients, 6% did not enrol in CR and did not attend the intake procedure at the outpatient clinic. Median waiting time (time between hospital discharge date and date of planned intake procedure) in the entire cohort was 11 days, without between-group differences for patients who enrolled or did not enrol. There were also no between-group differences in waiting time for patients who completed or did not complete CR (Tab. 1). Patients who did not enrol were older and were less often revascularised. The area under the ROC curve for this model was 0.76, indicating fair discriminatory ability (Fig. 2b).

### Predictors of completion

Of the patients enrolled in CR, 24% did not complete their CR programmes (i.e. dropped out). Patients who dropped out were older and more often had hypertension, diabetes mellitus and prior manifestations of CAD (Tab. 1). They also had worse exercise capacity at baseline, were less often employed and had a worse quality of life. Characteristics independently associated with dropout were older age and worse exercise capacity (Tab. 2). The area under the ROC curve for this model was 0.71, indicating fair discriminatory ability (Fig. 2c).

### Attendance at group-based and individual therapies

Of the 414 patients enrolled in CR, 47% attended the education sessions, 90% attended the exercise training programme, 9% attended relaxation therapy and 5% attended psychoeducative prevention therapy. Regarding individual therapies, 25% visited a dietician (at least one visit) and 8% visited a psychologist (at least one visit).

## Discussion

In this study, nonreferral was the most important reason for not completing a CR programme after AMI. One-third of patients were not referred, and although dropout after referral was lower, more than 50% of discharged patients ultimately did not complete a CR programme. We found older age, female sex, traveling distance, NSTEMI, no coronary revascularisation and prior manifestations of CAD to be independently associated with nonreferral, which is in line with previous studies [22, 23].

The reason why several subgroups of patients are referred less often is only partly understood. Several studies have suggested that some physicians believe women or elderly patients will benefit less from CR [24, 25]. For patients with NSTEMI or without coronary revascularisation, the perceived benefit of CR may also be lower. In this case, however, limited capacity at CR facilities or lack of reimbursement for specific subgroups may also lead to lower referral rates [24], although the available evidence provides no reason for not referring these subgroups [26]. For elderly patients, transport and mobility issues may influence CR participation or willingness to be referred, as do social obligations, providing informal care or a belief that they may not benefit from CR. Widespread implementation of fully automatic referral strategies (including a mandatory justification in case a patient is not referred) may reduce selective referral, leading to improved referral rates. Besides, education of both patients and healthcare professionals on the benefits of CR may improve their motivation and attitudes towards CR and may reduce prejudices that CR is less effective in certain subgroups.

The participation rate in our cohort was 58% (371 out of 640 eligible patients started one or more therapies), which is higher than it was in recent studies [8, 18]. This may be due to differences in the definition of participation and the methods of data collection (e.g., EHR or survey data), and the possibility that participation rates have increased over time. Completion rates in our population were comparable to those in previous studies, although different definitions of completion were used [10, 18]. We found older age and worse exercise capacity at baseline to be independently associated with CR dropout. Low exercise capacity may be caused by comorbidity, which we could only partially correct for in our analyses. However, as low exercise capacity is associated with worse prognosis [27], it is even more important for these patients to participate in a multidisciplinary secondary prevention programme.

Although it seems that improving referral should have priority when aiming to increase CR utilisation, optimisation of enrolment, adherence and completion should not be overlooked. A recent Cochrane review, however, has only found limited evidence for interventions improving participation in and adherence to CR [28]. Home-based CR or cardiac telerehabilitation (CTR) is a safe and (cost-)effective alternative to centre-based CR [29], and future research should indicate whether implementation of CTR indeed leads to increased participation and completion rates. Still, budget ceilings may limit this potential increase in CR utilisation, even with full reimbursement for CR. Updated economic analyses, such as those by Frederix [11] and De Gruyter [12], are therefore needed to convince health insurers and policy makers of the benefits and return on investment of CR.

### Strengths and limitations

This study is the first to assess CR participation and dropout in the entire care pathway from hospital discharge after AMI to CR completion. Another strength is the fact that our data reflect referral, enrolment and completion in actual practice instead of being part of a prospective trial, in which selection bias may occur.

However, due to the retrospective nature of our study and the use of CTcue, we could not register data on socioeconomic status, contraindications or physician endorsement for CR, and reasons for nonreferral or dropout. Second, data on cardiovascular risk factors could not be reliably collected for patients who were not referred; therefore, these data were not used in analyses evaluating referral.

## Conclusion

For patients discharged after AMI, the main reason for not participating in CR was nonreferral. To optimise CR utilisation, improvement of referral rates should have priority, focusing on elderly patients and women, and on patients with NSTEMI, without coronary revascularisation or with prior manifestations of CAD. To improve CR enrolment and completion, alternative models of CR, such as CTR, should be considered.
